# Identifying Key Factors of Hazardous Materials Transportation Accidents Based on Higher-Order and Multilayer Networks

**DOI:** 10.3390/e25071036

**Published:** 2023-07-10

**Authors:** Cuiping Ren, Bianbian Chen, Fengjie Xie

**Affiliations:** School of Modern Post, Xi’an University of Posts and Telecommunications, Xi’an 710061, China; believe.323@163.com (C.R.); chenbian727@163.com (B.C.)

**Keywords:** hazardous materials transportation, accidents causation network, higher-order network, multilayer network, weighted k-core decomposition

## Abstract

This paper focuses on the application of higher-order and multilayer networks in identifying critical causes and relationships contributing to hazardous materials transportation accidents. There were 792 accidents of hazardous materials transportation that occurred on the road from 2017 to 2021 which have been investigated. By considering time sequence and dependency of causes, the hazardous materials transportation accidents causation network (HMTACN) was described using the higher-order model. To investigate the structure of HMTACN such as the importance of causes and links, HMTACN was divided into three layers using the weighted k-core decomposition: the core layer, the bridge layer and the peripheral layer. Then causes and links were analyzed in detail. It was found that the core layer was tightly connected and supported most of the causal flows of HMTACN. The results showed that causes should be given hierarchical attention. This study provides an innovative method to analyze complicated accidents, which can be used in identifying major causes and links. And this paper brings new ideas about safety network study and extends the applications of complex network theory.

## 1. Introduction

Hazardous materials transportation safety, especially road transportation, is closely tied to people’s lives and property and getting a growing concern. For example, the National Roadway Safety Strategy, published by the U.S. Department of Transportation, proposed zero roadway fatalities and serious injuries [1]. The State Council of China printed the National Road Traffic Safety Plan for the “Fourteenth Five Year Plan” and put it forward to decrease fatalities and frequency of traffic accidents [2]. Road transportation of hazardous materials is considered to be the most dangerous to public health [3]. When it happened, nearby residents usually suffer the most. And its accident causes are complex by the coupling effect of many unsafe factors including humans, vehicles, the environment, etc., and the inherent danger of hazardous materials. At present, hazardous materials transportation accidents occurring on the road are maintaining a high frequency of growth [4,5]. For example, during the period 2013–2019, 2777 accidents involving the road transportation of dangerous goods occurred in China, except occurring in loading, unloading, storage and maintenance [5]. Therefore, it is urgent to curb these accidents.

Identifying key causes and relationships is quite important to prevent accidents. Accident causes often involve nonlinear relationships and complex dynamic processes. Various factors, such as human, equipment, vehicle, environment and management, interact with each other and increase the complexity of transportation accidents [6]. For example, the combination of heavy fog weather and fatigue driving can lead to serious accidents. However, existing studies on accident causation mostly analyzed the influence of single-factors without considering the interactions of multiple factors [7,8,9]. The popular methods of accident causation mainly contain sequence models, epidemiological models, and system models [10]. These models cannot fill in the above gaps yet. In recent years, a complex network has emerged as a valuable tool for studying accident causation [9,11,12]. It provides a framework for representing and analyzing the interconnected relationships between the various factors of accidents. Although these studies have provided valuable insights, the complex interdependencies and higher-order relationships that exist within the accident causation systems are often failed to capture [13]. This is due to the fact that complex network is usually built by pairwise models. The chain reactions of causal factors with sequences and dependencies are usually neglected [14], that is the next incident is closely related to previous incidents.

To address the above problems, this paper aims to investigate the application of higher-order networks and multilayer networks in identifying critical factors contributing to hazardous material transportation accidents. By considering the interactions and dependencies between different causes of accidents, the importance of causes and links can be obtained. Theoretically, a new idea for safety network study and an application of network theory was developed. In application, this paper proposes valuable insights into the underlying causes and references for accident control. The rest of this paper is organized as follows. Section 2 describes the related works. Section 3 describes the details of the data and methods. Section 4 presents the multilayer properties of HMTACN. Section 5 presents the discussion. Finally, we show the conclusions of our study.

## 2. Related Works

Accident causation models, traditional approaches to analyzing accidents, have formed a relatively mature theoretical system [10]. Sequence models are simple linear thinking statistical analyses, such as dominoes [15], which are only adapted to simple factor analysis. Epidemiological models emphasize the joint analysis of the causes at individual and organizational levels [7,16]. Jiang et al. [7] used Human Factors Analysis Classification System (HFACS) to classify the causes of hazardous chemical storage accidents and found that resource management was a significant cause of accidents, followed by violations and inadequate supervision. System models are more comprehensive in analyzing the hierarchical relationships among causal factors and complex nonlinear interactions [17,18]. Leveson [17] proposed System Theoretic Accident Model and Processes (STAMP) based on system theory in 2004. STAMP seeks to identify and analyze these complex interactions using a set of modeling techniques. Therefore, accident cause analysis remains a hot topic. However, the following gaps remain in these studies: (i) Sequence models may overlook nonlinear temporal relationships, epidemiological models may ignore heterogeneity among individuals, and system models may overlook complex interactions quantitalitively. These may not fully reflect the complex accident causation mechanisms in the real world. (ii) These lack quantitative research, which is not conducive to a larger number of accident causation systems. (iii) Lack of consideration of multi-factor interactions.

To expose the complex and dynamic relationships of a large number of accidents, a complex network has been applied to analyze accidents, such as railway accidents [8,11], subway accidents [9,19], road accidents [20] and production safety accidents [21], etc. Li et al. [8] proposed a risk monitoring model based on complex networks to quantify the risk of accident causal factors. Zhou et al. [9] used network theory to study the complexity of the subway construction accident network. Complex network theory provides a new idea for revealing important factors and functional mechanisms among factors. The pairwise relation model is used in complex networks, which assumes that the transfer of the node only depends on the current position. This illustrates the memoryless property of the Markov chain [13]. However, in a real complex system an accident chain usually requires more than two steps, and the dependency information cannot be described by a pairwise relation model [22]. Therefore, a complex network lacks information on the sequence and dependencies of causes, which affects the accuracy of key factors identification.

In relation to the problem, a higher-order network was proposed in 2016. Xu et al. [23] demonstrated that data from real-world systems can reveal fifth-order dependencies, and proposed a higher-order network (HON). Higher-order structures [24,25], such as hypergraphs, multilayer models, and simplex models are effective tools for describing complex relationships in the real world. In comparison with the traditional complex network, it can record the sources and destinations of nodes to make up for the shortcomings of the traditional theory in describing the coupling interaction relationships [23,26]. A higher-order network representation and algorithm were proposed to indicate that it has an important impact on the agglomeration, node ordering and scalability of the network [27]. Nowadays, scholars have demonstrated that the higher-order network is better than the traditional first-order network in identifying key nodes and revealing the role between nodes [28]. However, higher-order networks had been studied in the field of transportation [29], but not yet in analyzing accidents. Therefore, identifying key causes and links of hazardous materials transportation by higher-order networks is an important issue.

Although a higher-order network has advantages in constructing hazardous materials transportation accidents causation network, it lacks the ability to analyze structure such as the importance of causes and links. In fact, a multilayer network is applicable to analyze real situations, which can break the limitation of homogeneity in single-layer networks and identify more precisely how factors develop in each layer of a complex system [30,31]. However, most studies of accident causation networks had ignored their multilayer properties. Currently, multilayer networks had been used in the transportation field, such as airline networks [32,33], railroad networks [34] and public transportation networks [35,36], but rarely in accident causation analysis. Du et al. [33] divided the Chinese Airline Network (CAN) into three layers and analyzed its robustness. It was found that the CNA is more fragile when the core layer is not fully connected. Zhou et al. [19] established a subway construction safety risk network (SCSRN) based on the complex network and explored the correlation between accident reasons.

In summary, most existing research on hazardous materials transportation accidents analyzed the causes from a single-factor qualitative perspective, lacking consideration of multi-factor interactions. In addition, the dependencies and multilayer structure of the causes in hazardous materials accidents were not considered, resulting in imprecise identification of the causes. Higher-order and multilayer networks are able to fill these gaps. Therefore, this paper proposes the multi-factors and constructs an accident causation network with the characteristics of hazardous material transportation using a higher-order network. To analyze the importance of causes and their interactions, the structure is explored using a multilayer network. By integrating different findings and approaches, we can gain a more comprehensive understanding of accident causation.

## 3. Materials and Methods

### 3.1. Research Data

In this paper, Hazardous Materials Transportation Accidents (HMTAs) occurring on the road from 2017 to 2021 in China are investigated by the China Federation of Logistics & Purchasing (CFLP) and China Chemical Safety Association (CCSA). Finally, 792 accidents with more complete information are collected.

**Figure 1:**
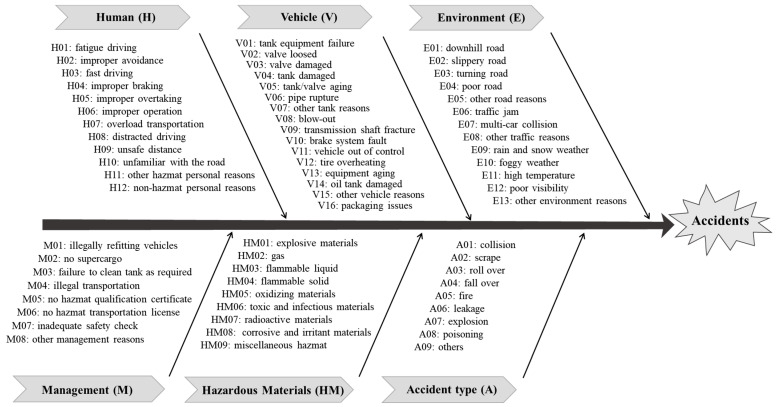
Accident causal factors.

Causal factors in HMTAs should be identified first. Based on the accident causation theory and characteristics of hazardous materials transportation, causal factors are divided into single-factors and multi-factors, and encoded with order numbers. With a systematic classification method and inspired by some studies about accidents [3,8,9,11,20,21], single-factors are unsafe factors from man, vehicle, hazardous materials, environment and management. Each type of single factor contains detailed causes. Also, considering influences of accident forms, 9 accident types are classified into single factors, which contain collision, scrape, roll over, fall over, fire, leakage, explosion, poisoning and others. Based on our previous research on hazardous materials transportation accidents [37], Figure 1 shows the single factors of HMTAs. Multi-factors emphasize the combined actions of two or more single-factors, for example, a multi-factor E2H6 means combined actions of E02 (slippery road) and H06 (improper operation). Ultimately, 129 accident causal factors, containing 67 single-factors and 62 multi-factors, are found. Then, the 792 accidents are transformed into accident chains, which corresponded to the codes. The specific process is illustrated by two examples, as shown in Table 1.

### 3.2. Modeling HMTACN

Since time sequence and dependence are key characteristics of HMTAs, this paper builds HMTACN with the idea of non-Markov chains in higher-order network theory. With HMTAs’ causal factors and the chains aforementioned, path dependency should be extracted, then a network can be constructed. Based on the BuildHON+ algorithm [27], the process of this network contains two steps. Step 1 is used to illustrate the path dependency extraction process and step 2 is used to illustrate the network construction process.

**Figure 2:**
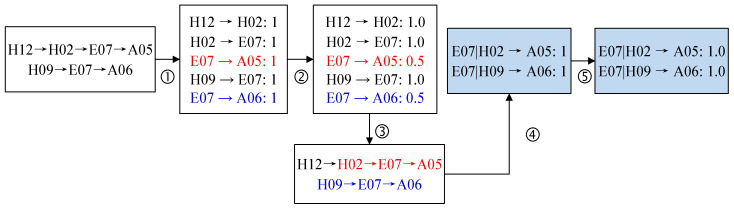
Path dependency extraction. E07 can point to A05 and A06, which are marked with red and bule respectively. The seconde-order sub-paths are marked with blue background.

Step 1: Path dependency extraction can be described with two paths (Figure 2). First, all first-order sub-paths are extracted from the two accident chains and the frequency of each sub-path is calculated in Figure 2①. Second, the transition probabilities of the first-order sub-paths H12 → H02, H02 → E07 and H09 → E07 are all 1.0 by Formula (1), indicating that the transition probabilities of these are determined. But E07 may go to A05 or A06 with frequency 1 in Figure 2②. According to $P=P\left(E07\to A05\right)=P\left(E07\to A06\right)=1/(1+1)=0.5$, the transition probabilities of E07 → A05 and E07 → A06 are both 0.5 with uncertain. Third, the first-order sub-paths E07 → A05 and E07 → A06 are derived from H02 and H09, respectively. Then they are extended to H02 → E07 → A05 and H09 → E07 → A06 in Figure 2③. Finally, the frequency of both second-order sub-paths is calculated as 1 in Figure 2④. Since $P=\left(E07\to A05\right)=P\left(E07\to A06\right)=1/1=1.0$, the dependency growth stops in Figure 2⑤. Thus, E07|H02 → A05 and E07|H09 → A06 form the second-order dependency.

(1) $$P_{ij}=P\left(i\to j\right)=\frac{W(i\to j)}{\sum_{h}W(i\to j)}\;$$

where, W(i→j) is the frequency from node i to j, and h denotes the set of nodes that node i may go at time t + 1.

**Figure 3:**
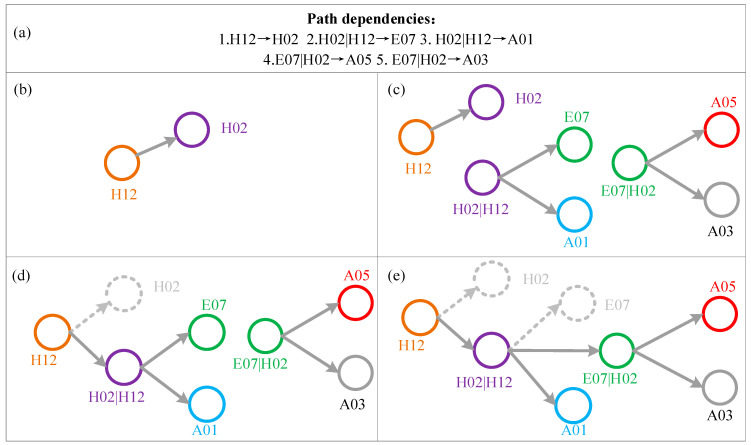
Network construction of HMTACN. Five path dependencies is listed in (**a**,**b**) shows first-order path dependency. (**c**) adds two the second-order nodes and their outgoings. Edges are reconnected to second-order nodes in (**d**,**e**).

Step 2: The network construction process is illustrated by an example of five path dependencies, shown in Figure 3. First, the first-order nodes and their corresponding edges are formed by converting the first-order path dependencies. As shown in Figure 3b, H12 → H02 is attached to a network and converted into H12 and H02. Second, the second-order node H02|H12 and two outgoing edges are formed by converting H02|H12 → E07 and H02|H12 → A01 in Figure 3c. Similarly, the second-order node E07|H02 and two outgoing edges are formed by E07|H02 → A05 and E07|H02 → A03. Finally, the edges of all higher-order nodes are reconnected. Node H02 is derived from H12 to form the second-order node H02|H12, and reconnects H12 → H02 to “H02|H12” in Figure 3d. Similarly, the edge H02|H12 → E07 is reconnected to the second-order node “E07|H02” in Figure 3e.

**Figure 4:**
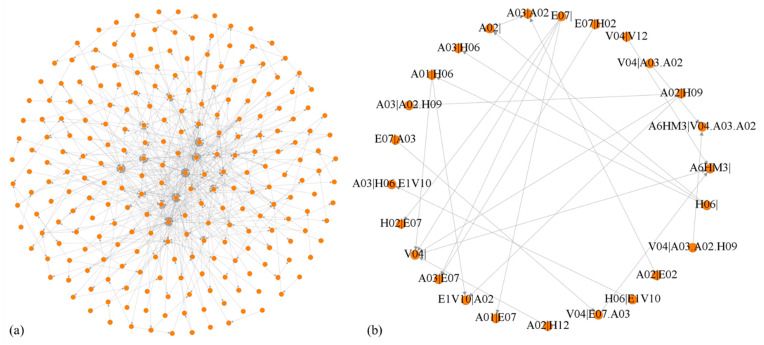
(**a**) The topology of HMTACN. (**b**) The partial topology of HMTACN.

Finally, HMTACN contains 243 nodes and 545 edges. Nodes represent causal factors with 124 first-order nodes and 119 higher-order nodes; the maximum order is four. Edges represent interrelationships of nodes. The HMTACN is visualized by Gephi, shown in Figure 4a. Gephi is an open-source network analysis and visualization software that provides a visual way to analyze complex networks. Figure 4b shows part of HMTACN, which clearly displays detailed interactions of factors. There are five first-order nodes (A02|, E07|, A6HM3|, H06| and V04|) representing causal factors and twenty higher-order nodes representing path dependencies. For example, the second-order node A01|H06 represents a second-order dependency with H06 → A01, and the third-order node V04|E07.A03 represents a path dependency with A03 → E07 → V04.

### 3.3. Measurements in HMTACN Analysis

#### 3.3.1. Degree and Strength

**Degree:** $k_{i}$

The degree of node i is the number of edges connected to it and is calculated as shown in Formula (2):

(2) $$k_{i}=\sum_{j=1}^{N}a_{i,j}$$

where:

*N*—the total number of nodes in the network;

$a_{i,j}$—$a_{i,j}$ = 1, there is an edge between node i and j, otherwise, $a_{i,j}=0$.

**Strength:** $S_{i}$

The strength of node i is the sum of the weights with the edges directly connected to it, which is called “accident causal flows” in this paper. It is calculated as shown in Formula (3):

(3) $$S_{i}=\sum_{j=1}^{N}a_{i,j}\;w_{i,j}$$

where:

$w_{i,j}$—the weights of the edges between node i and j.

#### 3.3.2. Weighted k-Core Decomposition

Since the weighted k-core decomposition was proposed by Antonios Garas et al. [38], it has often been applied in multilayer to analyze the multilayer structure of networks [33,34]. And the specific stratification depends on the study subjects and data characteristics. To further analyze the causal factors and reveal the multilayer characteristics, the weighted k-core decomposition is used to analyze HMTACN in this paper. The calculation is shown in Formula (4):

(4) $${k_{i}}^{\prime }={\left[{k_{i}}^{\alpha }{\left(\sum_{j}^{k_{i}}w_{i,j}\right)}^{\beta }\right]}^{\frac{1}{\alpha +\beta }}$$

where:

$k_{i}$—the degree of node i;

$w_{ij}$—the weight between node i and its neighboring j;

When the parameters α=β=1, the degree and weight have a similar influence.

The steps of the weighted k-core decomposition are as follows.

Step 1: Calculate the total degree $k_{i}$ of all nodes.

Step 2: Gradually remove nodes with $k_{i}=1$, which belongs to the same layer of the network.

Step 3: Gradually remove nodes with ${k_{i}}^{\prime }=1$, which may be completely disconnected from the main network.

Step 4: Remove the new nodes with ${k_{i}}^{\prime }<k_{i}$ and keep repeating this process until ${k_{i}}^{\prime }\geq k_{s}+1$ for all nodes in the network. Then mark the removed nodes as $k_{s}$ and $k_{s}=k_{s}+1$.

Step 5: The algorithm stops when all the nodes are marked with $k_{s}$.

#### 3.3.3. Measurements in HMTACN

In HMTACN, several nodes of different orders may correspond to one causal factor in accident chains. When the HMTACN is layered using the weighted k-core decomposition, nodes of different orders may be assigned to different layers. The following are the corresponding measurements.

(1) **Layer-degree:** $k_{il}$

The layer-degree of the node i is the sum of the degrees of all nodes of different orders corresponding to causal factor i in layer l. It is calculated as Formula (5).

(5) $$k_{il}=\sum_{m=1}^{M}k_{il}^{m}$$

It should be emphasized that HMTACN is a directed network, the layer-in degree and layer-out degree are proposed. The layer-in degree of node i is the sum of the entry degrees of all nodes of different orders corresponding to causal factor i in layer l. It is calculated as Formula (6).

(6) $${k_{il}}^{in}=\sum_{m=1}^{M}k_{il}^{m-in}$$

The layer-out degree of node i is the sum of the exit degrees of all nodes of different orders corresponding to causal factor i in layer l. It is calculated as Formula (7):

(7) $${k_{il}}^{out}=\sum_{m=1}^{M}k_{il}^{m-out}$$

where:

*M*—the total number of all nodes of different orders corresponding to node i in layer l;

$k_{il}^{m}$—the degree of the *m*th node corresponding to node i in layer l.

(2) **Layer-strength:** $S_{il}$

The layer-strength of the node i is the sum of the strengths of all nodes of different orders corresponding to causal factor i in layer l. It is calculated as Formula (8):

(8) $$S_{il}=\sum_{m=1}^{M}S_{il}^{m}$$

where:

$S_{il}^{m}$—the strength of the *m*th node corresponding to node i in layer l.

(3) **Layer-**ks**:** $k_{sil}$

The layer-$k_{s}$ of the node i is the sum of the $k_{s}$ of all nodes of different orders corresponding to causal factor i in layer l. It is calculated as Formula (9):

(9) $$k_{sil}=\sum_{m=1}^{M}k_{sil}^{m}$$

where:

$k_{sil}^{m}$—the $k_{s}$ of the *m*th node corresponding to node i in layer l.

(4) **The ratio of causal node connection and causal flow within and between the layers**

In order to deeply analyze network topology and node characteristics for different layers, the ratio of causal node connection and the ratio of causal flow are defined from intra-layer and inter-layer aspects, as shown in Table 2.

Where:

$N_{layer}$—the number of neighbors of node i within the layer, for example, $N_{core}$ means the number of neighbors in the core layer, $N_{bridge}$ means the number of neighbors in the bridge layer, and $N_{total}$ means the total number of neighbors of node i within and between the layers.

$F_{layer}$—the number of causal flows of node i with neighbors in the layer, for example, $F_{core}$ means the number of causal flows with the neighbors in the core layer of this causal factor, $F_{bridge}$ means the number of causal flows with the neighbors in the bridge layer of this causal factor, $F_{total}$ means the total number of causal flows that start or end within this causal factor.

${R_{a}}^{in}$—the ratio of causal nodes within the layers;

${R_{a}}^{out}$—the ratio of causal nodes between layers;

${R_{f}}^{in}$—the ratio of causal flow within the layers;

${R_{f}}^{out}$—the ratio of causal flow between the layers;

${R_{a}}^{cb}$—the ratio of causal node connection between the core layer and bridge layer, ${R_{a}}^{cp}$, ${R_{a}}^{bc}$, ${R_{a}}^{bp}$ are similar;

${R_{f}}^{cb}$—the ratio of causal flow between the core layer and bridge layer, ${R_{f}}^{cp}$,${R_{f}}^{bc}$,${R_{f}}^{bp}$ are similar.

## 4. The Multilayer Properties of HMTACN

### 4.1. Multilayered Structure of HMTACN

**Figure 5:**
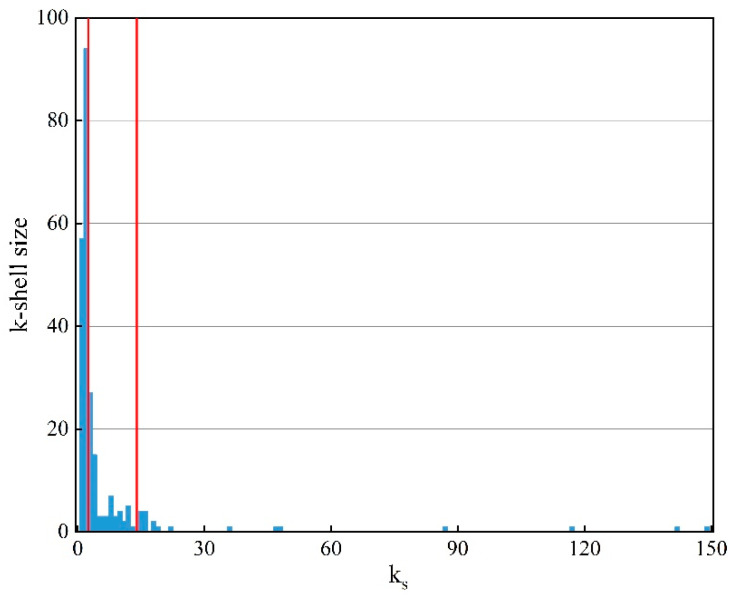
The result of the weighted k-core decomposition. k−shell size represents the number of nodes with $k_{s}$.

According to the weighted k-core decomposition mentioned in Section 3.3.2, the multilayer structure of HMTACN is proposed. Figure 5 shows the result of the layers.

Theoretically, the nodes corresponding to each $k_{s}$ can be used as a separate layer, but too many nodes are not conducive to the study. Inspired by the division of multilayer networks in the traffic domain [29,33,34], HMTACN can be divided into three layers: the core layer, the bridge layer and the peripheral layer by the distribution characteristics of $k_{s}$. As Figure 5 shows, the first gap appears and k−shell shows a continuous decreasing trend when $k_{s}$ ≥ 15. And the corresponding nodes within this range are assigned to the core layer, which indicates 19 nodes are the core nodes in HMTACN; when $k_{s}$ ≤ 2, the k−shell reach the maximum value, and the corresponding nodes are assigned to the peripheral layer; and when 3 ≤ $k_{s}$ < 15, these corresponding nodes are assigned to the bridge layer.

**Figure 6:**
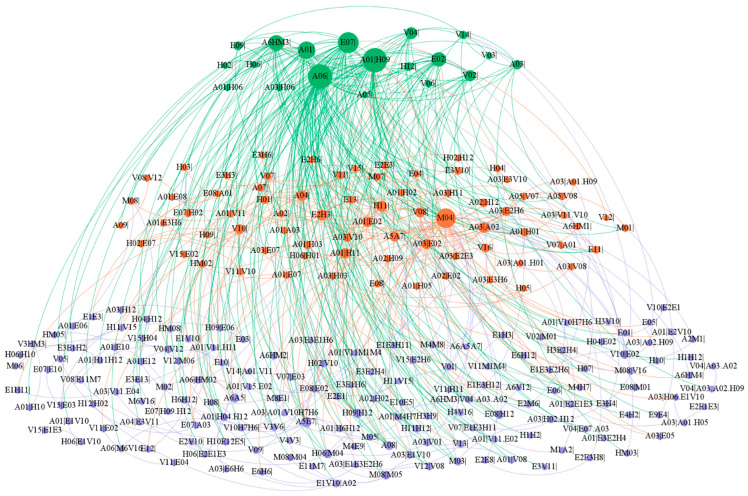
The multilayer structure of HMTACN.

The multilayer structure of HMTACN is shown in Figure 6 by Gephi. The node size represents the number of factors connected to it. Here green represents the core layer, orange represents the bridge layer, and purple represents the peripheral layer. The color of the edge is the color of the target node. Since the existence of higher-order nodes in the multilayer structure of HMTACN, one cause may correspond to several different nodes and be assigned to different layers. Also, multiple nodes in a layer may correspond to a cause. For example, H02 corresponds to the H02|H12, H02|E07 and H02|V10 in HMTACN, where H02 is part of the core layer, H02|H12 and H02|E07 are part of the bridge layer and H02|V10 is part of the peripheral layer. Therefore, node H02 belongs to three layers. It is found that 19 causal factors belong to two layers accounting for 15.32%, and 6 causal factors belong to three layers, accounting for 4.84%.

In different layers, the nodes and edges present different characteristics, as shown in Table 3.

In the core layer, there are 16 causal factors. The value of $\bar{S_{il}}$ has a wide range from 34 to 846. Actually, 14 causal factors have an average layer strength between 34 and 247, and 2 causal factors have much higher $\bar{S_{il}}$ with 704 and 846, respectively. Obviously, the core layer has the largest $\bar{k_{sl}}$ value. In addition, 70 edges in the core layer bear 1511 causal associations, with an average of 21.59 per edge, accounting for 63% of the network. In the bridge layer, there are 40 causal factors and the value of $\bar{S_{il}}$ have a small variation, from 3 to 37. The 67 edges in the bridge layer bear 143 causal associations, with an average of 2.13 per edge. There are 98 causal factors in the peripheral layer and the value of $\bar{S_{il}}$ is only between 1 and 5, indicating that each causal factor in the peripheral layer appears less frequently. The 57 edges in the peripheral layer bear 61 causal associations, with an average of 1.07 per edge. The edges between the three layers can reflect the inter-layer relationship of causal factors. Clearly, the relationship between the core and bridge layers is prominent in Table 3.

### 4.2. Importance of Causes and Links in HMTACN

#### 4.2.1. Importance of Causes in HMTACN

As aforementioned, HMTACN is divided into the core layer, bridge layer and peripheral layer based on $k_{s}$. The $k_{s}$ value of a node can reflect the number of connected nodes, the frequency of related edges and the ability to connect other layers. Usually, the larger the $k_{s}$ is, the more important the node is. Therefore, causes are classified into seven levels (I–VII) by dividing the nodes within different layers, shown in Table 4.

In Table 4, there are 5 causes in level I with nodes belonging to core, bridge and periphery layers simultaneously. And the $\bar{k_{s}}$ value in level I is the largest. This indicates the causes in level I are the most important causal factors in HMTACN. Among them, the three higher-order nodes that represent dependencies A01|H09, A01|H06 and A03|H06 belong to the core layer, which cannot be captured by traditional complex networks. In level II, only A05 belongs to core and bridge layers and its $\bar{k_{s}}$ value is 42. However, in level III, the $\bar{k_{s}}$ value is 68.83 which is relatively larger than level II. This is because the $k_{s}$ value of V04 is 157 and V06 is 145 which increases the average $k_{s}$ value in level III. In levels I, II, III and IV, the $\bar{k_{s}}$ value is large and node number is small relatively. Therefore, the 16 causes within levels I to IV are core causal factors in the HMTAC system.

From level V to level VII, $\bar{k_{s}}$ values decrease and node numbers increase successively. Further, the causes in level V are mainly vehicle factors, and nodes in level VI cover almost all causal factor types. In level VII, multi-factors account for 71.62%, and 47.17% are related to environmental factors.

#### 4.2.2. Importance of Links in HMTACN

The links importance of HMTACN is measured by $\bar{w_{l}}$ (the average weight of the edges in layer l) shown in Table 5. The greater $\bar{w_{l}}$, the greater accident causal flow, the more important the link is.

As shown in Table 5, at level I, the values of $\bar{w_{l}}$ is the most with 70 links accounting for 12.84%. Thus, it is important to focus on these links. From level II, the values of $\bar{w_{l}}$ decrease sharply. This means these links undertake fewer cause connections. At level II, the number of links is the largest, accounting for 32.29%. Level III has fewer links and lower weights. The values of $\bar{w_{l}}$ from levels IV to VI are similar, while the number at level IV is much larger than at levels V and VI. This indicates most of the bridge and peripheral layer causes tend to connect with the core layer, while the links between the bridge and peripheral layer are sparse.

In order to reveal the significant links of HMTACN, the study explores the links within level I, shown in Table 6. In the type of vehicle causes, V04, V03 and V02 are easily leading to A06 accidents. Especially, the link frequency of V04 → A06 is the highest, accounting for 24.42%. In the type of accident causes, A01 → V04 is the most important link, the value of weight is 216. In addition, A03 is easily leading to V04 and A06. In the type of human cause, H12 → A01 is clearly the most important link, the value of weight is 210. In addition, H02 and H06 are easily leading to A03. In the type of environmental causes, the weights of E09 → E02, E07 → V04 and E02 → E07 are similar and smaller, indicating that the environmental cause alone is not the most important causal factor.

### 4.3. Analysis of Causes and Links in Different Layers

#### 4.3.1. Core Layer

The core layer is the crucial layer in HMTACN. To discover the characteristics of the core layer, 16 causal factors are further analyzed by calculating the ratio of causal node connection and causal flow within the core layer and between the core layer and other layers. Table 7 shows the detailed calculations with ${R_{a}}^{in}$, ${R_{a}}^{out}$, ${R_{a}}^{cb}$, ${R_{a}}^{cp}$, ${R_{f}}^{in}$, ${R_{f}}^{out}$, ${R_{f}}^{cb}$, ${R_{f}}^{cp}$, which have been defined in Section 3.3.3.

As shown in Table 7, accident types acting as intermediaries remain larger $K_{il}$ in core layer, including A03 (roll over), A01 (collision), A06 (leakage) and A05 (fire). This indicates that these accidents occur most frequently and connect more factors, especially roll over, collision and leakage of hazardous materials. The unsafe status of the vehicle is relatively more in the core layer, including V04 (tank damaged), V03 (valve damaged), V02 (valve loosed), V06 (pipe rupture) and V14 (oil tank damaged), which mainly focus on equipment loading hazardous materials. It indicates that they connect more factors and the damage to vehicle facilities are key reasons in the core layer, especially V04. Unsafe behaviors of humans, like H12 (non-hazmat personal reasons), H02 (improper avoidance) and H06 (improper operation) are the main causes, especially H12. Also, environmental causes like E07 (multi-car collision), E02 (slippery road) and E09 (rain and snow weather) are important factors. In addition, multi-factors are significant similarities. For example, A6HM3 (leakage and flammable liquid) is most likely to cause fire and explosion.

**Figure 7:**
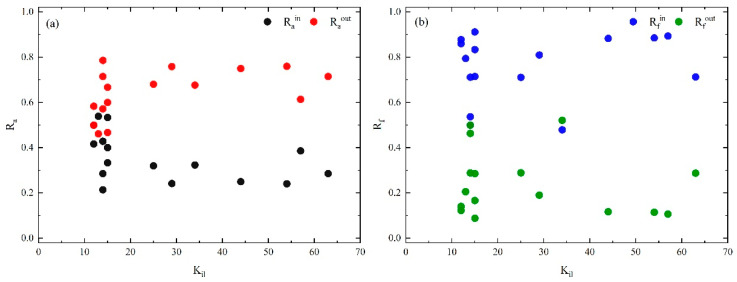
(**a**) ${R_{a}}^{in}$ and ${R_{a}}^{out}$ of casual factors in core layer vs. layer-degree. (**b**) ${R_{f}}^{in}$ and ${R_{f}}^{out}$ of casual flows in core layer vs. layer-degree within the layer.

**Figure 8:**
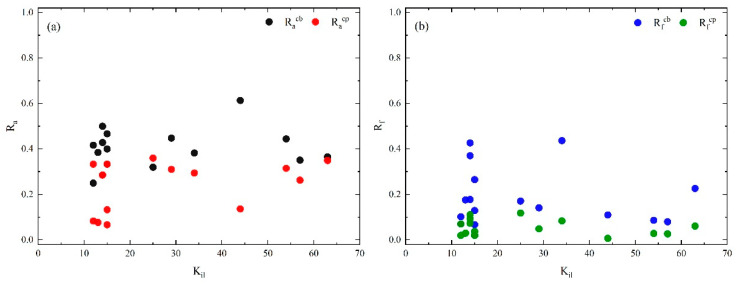
(**a**) ${R_{a}}^{cb}$ and ${R_{a}}^{cp}$ of casual factors in core layer vs. layer-degree. (**b**) ${R_{f}}^{cb}$ and ${R_{f}}^{cp}$ of casual flows in core layer vs. layer-degree within the layer.

To visualize the characteristics of the core layer, calculations of Table 6 are drawn in Figure 7 and Figure 8.

Figure 7 shows that $R_{a}$ and $R_{f}$ are not direct relation to the node higher-order degree ($k_{il}$) in the core layer. Except for E09 (rain and snow weather), A05 (fire) and V03 (valve damaged), the causal factors show that ${R_{a}}^{in}$ is much smaller than ${R_{a}}^{out}$, but ${R_{f}}^{in}$ is much larger than ${R_{f}}^{out}$. This suggests that interactions between causal factors within the core layer are more likely to lead to accidents than with the bridge and periphery layers. In addition, E09 and A05 show that ${R_{a}}^{in}$ > ${R_{a}}^{out}$ and ${R_{f}}^{in}$ > ${R_{f}}^{out}$, indicating that they tend to interact with causal factors in the core layer. It is worth noting that only the V03 ${R_{a}}^{in}$ < ${R_{a}}^{out}$ but ${R_{f}}^{in}$ < ${R_{f}}^{out}$ indicates that it is more closely connected to other layers. This can be attributed to both causal factors of E11 (high temperature) and E13 (other environmental reasons) in the bridge layer that are more likely to cause valve damage. It is found that the core layer has the strongest effect in HMTACN.

Figure 8 shows that the causal factors have the characteristics of ${R_{a}}^{cp}$ < ${R_{a}}^{cb}$ and ${R_{f}}^{cp}$ < ${R_{f}}^{cb}$ on the whole, indicating that the interaction between the core layer and the bridge layer is stronger than that between the periphery layer. Further, there are 4 edges with strength greater than 10 between the core layer and bridge layer, and 4 higher-order nodes that represent dependencies are generated: A01|H02, A03|E02, A01|A03, A01|E02. This illustrates two findings. Firstly, H02 (improper avoidance) → A01 (collision), E02 (slippery road) → A03 (roll over), A03 (roll over) → A01 (collision), E02 (slippery road) → A01 (collision) are the crucial causal links between the core layer and bridge layer. Similar findings have been found in previous studies [4]. Secondly, the dependency may have been missed by previous studies using complex networks. Such as A01|H02, collision usually depends on the generation of improper avoidance leading to chain accidents.

#### 4.3.2. Bridge Layer

**Figure 9:**
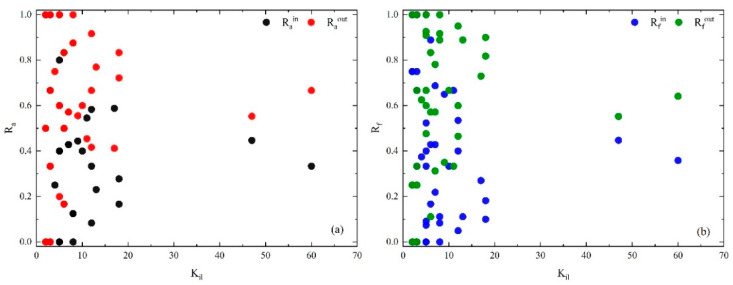
(**a**) ${R_{a}}^{in}$ and ${R_{a}}^{out}$ of casual factors in bridge layer vs. layer-degree. (**b**) ${R_{f}}^{in}$ and ${R_{f}}^{out}$ of casual flows in bridge layer vs. layer-degree within the layer.

**Figure 10:**
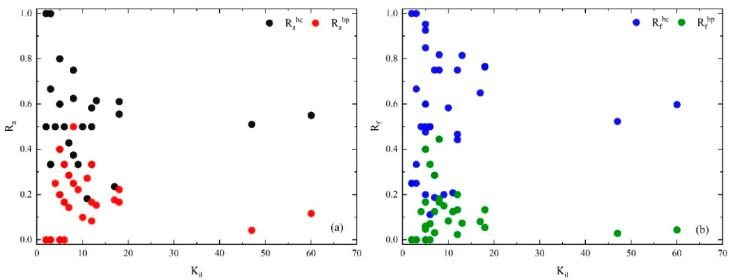
(**a**) ${R_{a}}^{cb}$ and ${R_{a}}^{cp}$ of casual factors in bridge layer vs. layer-degree. (**b**) ${R_{f}}^{cb}$ and ${R_{f}}^{cp}$ of casual flows in bridge layer vs. layer-degree within the layer.

Similar to the core layer analysis, Figure 9 shows the ratios within the bridge layer and between the bridge layer and the other two layers. In the bridge layer, most of the causal factors show ${R_{a}}^{in}$ < ${R_{a}}^{out}$ and ${R_{f}}^{in}$ < ${R_{f}}^{out}$, indicating that the bridge layer is more inclined to interact with outside the layer. Also, most causal factors in the bridge layer have the characteristics of ${R_{a}}^{bc}$ > ${R_{a}}^{bp}$ and ${R_{f}}^{bc}$ > ${R_{f}}^{bp}$ on the whole (in Figure 10), indicating that they tend to interact with the core layer.

To further explore important characteristics of causal factors in the bridge layer, these top 14 causal factors with larger ${R_{a}}^{bc}$ have been analyzed, shown in Table 8. These 14 causal factors have 76 edges with the core layer, accounting for 59.38% of all edges between the bridge layer and core layer. In addition, the value of ${R_{a}}^{out},{R_{f}}^{out},$ ${R_{a}}^{bc}$ and ${R_{f}}^{bc}$ are much larger than 0.5. Except for E13 (other environmental reasons) and E2H1 (slippery road and fast driving), M07 (inadequate safety check and maintenance) has the largest ${R_{a}}^{bc}$, followed by M08 (other management reasons). Causal factors connecting to M07 and M08 are V02 (valve loosed), V03 (valve damaged), V04 (tank damaged), V06 (pipe rupture), A05 (fire) and A06 (leakage), indicating that M07 and M08 need only one step to interact with these causal factors. But other causes require two or more steps. This indicates that the influence of the management factor is greater and the problem is more serious. In short, the interaction between the bridge layer and core layer is far closer than the periphery layer.

In the bridge layer, the value of $\bar{S_{il}}$ is less than 28 for most causal factors, except V16 (packaging issues), V12 (tire overheating), and H09 (unsafe distance). Therefore, these causal factors have been analyzed furthermore in Table 9. It is found that V16 only belongs to the bridge layer and has the largest value of $\bar{K_{il}}$ and $\bar{S_{il}}$ in the bridge layer, so the packaging issue is the most significant causal factor in the bridge layer. Moreover, only node V16 has a higher ${K_{il}}^{in}$ than ${K_{il}}^{out}$, which indicates that many causal factors can lead to the packaging issues, such as H04 (improper braking), A03 (roll over), A01 (collision), V15 (other vehicle reasons) and E04 (poor road). In addition, the value of ${R_{f}}^{out}$ and ${R_{f}}^{bc}$ of V16, V12 and H09 are relatively larger, which illustrates that they are more closely connected with the core layer.

#### 4.3.3. Periphery Layer

**Figure 11:**
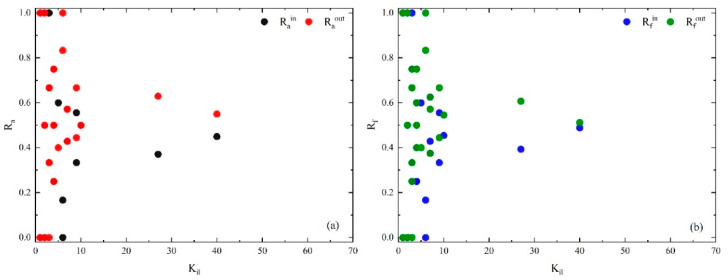
(**a**) ${R_{a}}^{in}$ and ${R_{a}}^{out}$ of casual factors in periphery layer vs. layer-degree. (**b**) ${R_{f}}^{in}$ and ${R_{f}}^{out}$ of casual flows in periphery layer vs. layer-degree within the layer.

Similar to the core layer analysis, Figure 11 shows the ratio within the peripheral layer and between the periphery layer and the other two layers.

As shown in Figure 11, the trends of $R_{a}$ and $R_{f}$ in the periphery layer are basically the same without obvious patterns. This is because these causal factors within the periphery layer appear less frequently in accident chains and most of them are multi-factors. Moreover, 68% of multi-factors are related to environmental factors. This explains that environmental factors often work in conjunction with other causal factors to cause accidents, so the abnormal environment should be alerted in time.

Furthermore, there are 44 causal factors with ${R_{a}}^{out}$ = ${R_{f}}^{out}$ = 1 in the periphery layer, which illustrates that these causal factors are only correlated with other layers. And among them, 31 causal factors only belong to the periphery layer, 19 of them are multi-factors, and most of them interact with E01 (downhill road), E02 (slippery road) and E03 (turning road).

## 5. Discussion

In this study, the method combining higher-order and multilayer networks is applied to the field of hazardous materials transportation accidents for the first time. A total of 792 accidents occurring on roads from 2017 to 2021 are analyzed. The hazardous materials transportation accident causation network (HMTACN) is constructed using a higher-order network and divided into three layers using weighted k-core decomposition: the core layer, bridge layer, and peripheral layer.

As a result, 16 key causes covering five types of causes are identified through the analysis of the core layer. In the bridge layer, the management factors including M07 (inadequate safety check and maintenance) and M08 (other management reasons), the vehicle factors including V16 (packaging issues) and V12 (tire overheating), the human factor including H09 (unsafe distance) are critical causes of accidents. The analysis of the peripheral layer indicated that environmental factors often contributed to accidents in conjunction with other factors. The important results of each layer will be discussed from the perspective of several cause types, including vehicle, human, environment, accident types and management factors.

(i) Vehicle factors such as V04 (tank damaged), V03 (valve damaged), V02 (valve loosed), V06 (pipe rupture) and V14 (oil tank damaged) contribute to hazardous material accidents, as the damage to vehicle facilities can lead to leaks and increase the probability of accidents. These factors are associated with the maintenance condition, aging, and manufacturing quality of the vehicles. Therefore, it is necessary to implement regular maintenance programs to ensure the integrity of vehicle tanks, valves, and pipelines. Also, promptly repair or replace any damaged components to prevent leaks, and strengthen quality control measures during the manufacturing process to ensure vehicles are built to high standards. In addition, V16 (packaging issues) and V12 (tire overheating) are also important causes of accidents. This is consistent with the findings of Ma et al. [39] that packaging problems are an important cause of hazardous materials transportation accidents. Therefore, the packaging of hazardous materials should be improved.

(ii) Human factors, including H12 (non-hazmat personnel reasons) by non-hazmat personnel, H02 (improper avoidance), H06 (improper operation) and H09 (unsafe distance) by hazardous material transport personnel, have a high likelihood of causing accidents. These are consistent with the findings of Oggero et al. [40] that valve failure in machinery and improper human operation are the major causes of accidents. Human factors are influenced by driver experience, training, awareness, and attitude. Therefore, developing comprehensive training programs for both non-hazardous personnel and hazardous material transport personnel is important. Also, it is necessary to emphasize emergency response protocols and defensive driving techniques and implement stringent screening and qualification processes to ensure that personnel have the appropriate competencies.

(iii) Environmental factors such as E07 (multi-car collision), E02 (slippery road) and E09 (rain and snow weather) often work in conjunction with other factors to cause hazardous material accidents. Poor road conditions increase the probability of human error. Therefore, it is necessary to establish effective communication channels to provide real-time weather updates and road condition alerts to drivers.

(iv) Different accident types, such as A03 (roll over), A01 (collision), A06 (leakage) and A05 (fire), have different causes and potential impacts. For example, roll over can cause traffic congestion and road blockages, collisions can result in vehicle damage and personal injuries, leakage can lead to environmental pollution and health risks, and fire can trigger explosions. Therefore, developing and enforcing strict safety standards covering the handling, storage and transportation of hazardous materials is crucial.

(v) Management factors play a significant role in HMTACN, and the lack of management leads to an increased probability of all factors and accidents [20]. Therefore, it is necessary to implement comprehensive safety checks, maintenance and quality control measures to ensure the integrity of vehicles and their components.

## 6. Conclusions

In conclusion, this study proposes a new accident causation method that combines higher-order and multilayer networks. Unlike previous methods, this approach considers the interactions and dependencies of factors, allowing for more accurate identification of key causes by considering the interactions and dependencies of factors. The method also conducts a quantitative analysis of complex accident causation systems, particularly when dealing with a large amount of accident data. Based on the findings, recommendations are discussed to mitigate the risks of hazardous material road transportation accidents. Implementing these measures can help reduce accidents and enhance the safety of hazardous material transportation.

According to this study, the significant causes and links should be given hierarchical attention due to their different roles in the hazardous materials transportation accident causation network. Overall, this study contributes to the understanding of hazardous material transportation accidents and provides valuable insights for developing effective preventive measures.

This paper considered the static indicators of hazardous materials transportation accidents causation network. Further research should focus on analyzing the complex interactions and propagations of causes and links. Additionally, the application of surrogate safety measures, such as traffic conflict analysis, for dynamic safety evaluation of hazardous materials transportation deserves further study. In the future, the major causes and relationships may be changed, so more detailed and updated dynamic data on hazardous materials transportation accidents should be studied continuously.

## Figures and Tables

**Table 1:** Two cases of accidents.

No.	Description of Accident	Relationship	Accident Chain
1	1 January 2021, in Panjin Dawei District, a tanker carrying 28 tons of oil products was driving normally. Suddenly, from the left side of the road, a car appeared crossing the road. The tanker could not avoid it, then hit the opposite lane of trucks. Finally, three cars burst into flames.	Non-hazmat personal reasons → improper avoidance → multi-car collision → fire	H12 → H02 → E07 → A05
2	At 2:00 a.m. on 9 May 2018, two cars driving on Provincial Road 321 with close distance, which lead to collisions of cars. This violent collision led to the leakage of concentrated sulfuric acid from a hazardous materials vehicle.	Unsafe distance → multi-car collision → leakage	H09 → E07 → A06

**Table 2:** The ratio of causal node connection and causal flow within and between the layers.

	The Ratio of Causal Node Connection		The Ratio of Causal Flow	
Intra-layer	${R_{a}}^{in}=N_{layer}/N_{total}$	(10)	${R_{f}}^{in}=$ $F_{layer}/F_{total}$	(11)
Inter-layer	${R_{a}}^{out}=1-{R_{a}}^{in}$	(12)	${R_{f}}^{out}=1-{R_{f}}^{in}$	(13)
	${R_{a}}^{cb}=N_{bridge}/{(N}_{total}-N_{core})$	(14)	${R_{f}}^{cb}=F_{bridge}/{(F}_{total}-F_{core})$	(15)
	${R_{a}}^{cp}=1-{R_{a}}^{cb}$	(16)	${R_{f}}^{cp}=1-{R_{f}}^{cb}$	(17)
	${R_{a}}^{bc}=N_{core}/{(N}_{total}-N_{bridge})$	(18)	${R_{f}}^{bc}=F_{core}/{(F}_{total}-F_{bridge})$	(19)
	${R_{a}}^{bp}=1-{R_{a}}^{bc}$	(20)	${R_{f}}^{bp}=1-{R_{f}}^{bc}$	(21)

**Table 3:** Detailed information on nodes and edges in different layers.

Layer	Number of Nodes	Number of Causes	$\bar{k_{sl}}$	$\bar{S_{il}}$	Number of Edges	$\bar{w_{l}}$
Core	19	16	43.53	34–846	70	21.59
Bridge	73	40	5.66	3–37	67	2.13
Periphery	151	98	1.62	1–5	57	1.07
Core-bridge	-	-	-	-	176	2.82
Core-periphery	-	-	-	-	114	1.10
Bridge-periphery	-	-	-	-	61	1.03

**Table 4:** Causes importance measurement. P represents the proportion of nodes at different levels.

Layers of Causes	$\bar{k_{s}}$	Level	Number	*P* (%)	Examples of Causes
Belonging to core, bridge and periphery layers	102.6	I	5	4.03	H02, H06, E07, A01, etc.
Only belonging to core and bridge layers	42	II	1	0.81	A05
Only belonging to core and periphery layers	68.83	III	6	4.84	H12, V04, A06, A6HM3, etc.
Only belonging to core layer	24.5	IV	4	3.23	E02, E09, V03, V06
Only belonging to bridge and periphery layers	15.62	V	13	10.48	H09, V07, M08, A02, etc.
Only belonging to bridge layer	5.14	VI	21	16.94	H01, V16, E04, A6HM1, etc.
Only belonging to periphery layer	1.46	VII	74	59.68	H07, V01, A5E7, E1H3, etc.

**Table 5:** Links importance measurement.

Layers of Causal Links	$\bar{w_{l}}$	Level	Number	Examples of Causal Links
The core layer	21.59	I	70	H02 → A03, A01|H04-V06, etc.
Between the core and the bridge layer	2.82	II	176	E02 → A04, A3|H06 → V07, etc.
The bridge layer	2.13	III	67	E04 → V16, E07|H02 → A03|E07, etc.
Between the core and the periphery layer	1.10	IV	114	A01|E06 → V03, A01|H6H12 → V05, etc.
The periphery layer	1.07	V	57	A03|A02.H09 → V04|A03.A02.H09, etc.
Between the bridge and the periphery layer	1.03	VI	61	A03|E02 → E07|A03, etc.

**Table 6:** The important links within the core layer by causal type. P represents the proportion of weight (w) of the edge in the core layer.

Cause Type	Links	Specific	*w*	*P* (%)
Vehicle	V04 → A06	tank damaged → leakage	369	24.42
	V03 → A06	valve damaged → leakage	92	6.09
	V02 → A06	valve loosed → leakage	36	2.38
Accident	A01 → V04	collision → tank damaged	216	14.30
	A03 → V04	roll over → tank damaged	98	6.49
	A03 → A06	roll over → leakage	49	3.24
Human	H12 → A01	non-hazmat personal reasons → collision	210	13.90
	H02 → A03	improper avoidance → roll over	27	1.79
	H06 → A03|H06	improper operation → roll over	20	1.32
Environment	E09 → E02	rain and snow weather → slippery road	25	1.65
	E07 → V04	multi-car collision → tank damaged	17	1.13
	E02 → E07	slippery road → multi-car collision	7	0.46

**Table 7:** Characteristics of the 16 causal factors in the core layer.

Type	Factor	$K_{il}$	${R_{a}}^{in}$	${R_{a}}^{out}$	${R_{a}}^{cb}$	${R_{a}}^{cp}$	${R_{f}}^{in}$	${R_{f}}^{out}$	${R_{f}}^{cb}$	${R_{f}}^{cp}$
Accident	A03	63	0.286	0.714	0.365	0.349	0.712	0.288	0.226	0.061
	A01	57	0.386	0.614	0.351	0.263	0.893	0.107	0.080	0.027
	A06	54	0.241	0.759	0.444	0.315	0.885	0.115	0.087	0.028
	A05	34	0.324	0.676	0.382	0.294	0.479	0.521	0.437	0.084
Vehicle	V04	44	0.250	0.750	0.614	0.136	0.883	0.117	0.110	0.007
	V03	29	0.241	0.759	0.448	0.310	0.810	0.190	0.141	0.049
	V02	25	0.320	0.680	0.320	0.360	0.711	0.289	0.171	0.118
	V06	15	0.400	0.600	0.467	0.133	0.833	0.167	0.130	0.037
	V14	13	0.538	0.462	0.385	0.077	0.794	0.206	0.176	0.029
Human	H12	15	0.333	0.667	0.333	0.333	0.911	0.089	0.068	0.021
	H02	14	0.214	0.786	0.500	0.286	0.537	0.463	0.370	0.093
	H06	12	0.500	0.500	0.417	0.083	0.878	0.122	0.102	0.020
Environment	E07	15	0.533	0.467	0.400	0.067	0.714	0.286	0.265	0.020
	E02	14	0.286	0.714	0.429	0.286	0.500	0.500	0.426	0.074
	E09	14	0.429	0.571	0.286	0.286	0.711	0.289	0.178	0.111
Multi-factor	A6HM3	12	0.417	0.583	0.250	0.333	0.860	0.140	0.070	0.070

**Table 8:** Characteristics of the top 14 causal factors of ${R_{a}}^{bc}$ ranking in the bridge layer.

**Factor**	$K_{il}$	${R_{a}}^{in}$	${R_{a}}^{out}$	${R_{f}}^{in}$	${R_{f}}^{out}$	${R_{a}}^{bc}$	${R_{a}}^{bp}$	${R_{f}}^{bc}$	${R_{f}}^{bp}$
E2H1	3	0.000	1.000	0.000	1.000	1.000	0.000	1.000	0.000
E13	2	0.000	1.000	0.000	1.000	1.000	0.000	1.000	0.000
M07	5	0.000	1.000	0.000	1.000	0.800	0.200	0.952	0.048
M08	8	0.000	1.000	0.000	1.000	0.750	0.250	0.818	0.182
A6HM1	3	0.333	0.667	0.333	0.667	0.667	0.000	0.667	0.000
H04	8	0.125	0.875	0.083	0.917	0.625	0.250	0.750	0.167
V15	13	0.231	0.769	0.111	0.889	0.615	0.154	0.815	0.074
V07	18	0.167	0.833	0.100	0.900	0.611	0.222	0.767	0.133
E11	5	0.000	1.000	0.000	1.000	0.600	0.400	0.600	0.400
M01	5	0.000	1.000	0.000	1.000	0.600	0.400	0.600	0.400
E3H3	5	0.400	0.600	0.074	0.926	0.600	0.000	0.926	0.000
A07	12	0.083	0.917	0.050	0.950	0.583	0.333	0.750	0.200
A02	18	0.278	0.722	0.182	0.818	0.556	0.167	0.764	0.055
A01	60	0.333	0.667	0.358	0.642	0.550	0.117	0.597	0.044

**Table 9:** Characteristics of the three largest factors of $\bar{S_{il}}$ in the bridge layer.

**Factor**	$\bar{K_{il}}$	$\bar{S_{il}}$	${K_{il}}^{in}$	${K_{il}}^{out}$	${R_{a}}^{in}$	${R_{a}}^{out}$	${R_{f}}^{in}$	${R_{f}}^{out}$	${R_{a}}^{bc}$	${R_{a}}^{bp}$	${R_{f}}^{bc}$	${R_{f}}^{bp}$
V16	17	37	13	4	0.588	0.412	0.270	0.730	0.235	0.176	0.649	0.081
V12	5	33	2	3	0.400	0.600	0.091	0.909	0.200	0.400	0.848	0.061
H09	7	32	3	4	0.429	0.571	0.219	0.781	0.429	0.143	0.750	0.031

## Data Availability

Not applicable.
